# Effects of Clarified Açaí Supplementation and Photobiomodulation on the Parotid Glands of Rats Submitted to Chemotherapy

**DOI:** 10.1111/jop.70072

**Published:** 2025-10-24

**Authors:** Wallacy Watson Pereira Melo, Walessa Alana Bragança Aragão, Maria Karolina Martins Ferreira, Vinicius Ruan Neves dos Santos, Leonardo Oliveira Bittencourt, Paulo Fernando Santos Mendes, Deborah Ribeiro Frazão, José Mário Matos‐Sousa, Hadassa Helez Neves Ferreira, José Messias Perdigão, Hannah Gil de Farias Morais, Herve Rogez, Roseana de Almeida Freitas, Manoela Domingues Martins, Rafael Rodrigues Lima, Renata Duarte de Souza‐Rodrigues

**Affiliations:** ^1^ Laboratory of Functional and Structural Biology, Institute of Biological Sciences Federal University of Pará Belém Pará Brazil; ^2^ Center for Valorization of Amazonian BioactiveCompounds, College of Biotechnology Federal University of Pará Belém Pará Brazil; ^3^ Department of Oral Pathology Federal University of Rio Grande do Norte Natal Rio Grande do Norte Brazil; ^4^ Department of Oral Pathology, School of Dentistry Federal University of Rio Grande do Sul Porto Alegre Rio Grande do Norte Brazil

**Keywords:** antineoplastic drugs, *Euterpe oleracea* Mart., parotid salivary gland, photobiomodulation

## Abstract

**Background:**

The antineoplastic drugs used in anticancer treatment regimens can induce changes in normal tissues, including salivary glands. This study evaluated the effects of clarified açaí and photobiomodulation (PBM) on the oxidative biochemistry and microstructure of the parotid glands of rats with oral mucositis.

**Methods:**

Male rats (*n* = 54) were divided into five groups: Negative control (no mucositis); Positive control (mucositis without treatment); PBM; Clarified açaí; and PBM + clarified açaí. Oral mucositis was induced by intraperitoneal injection of 5‐fluorouracil on days 0 (60 mg/kg) and 2 (40 mg/kg). On days 3 and 4, bilateral scarification of the buccal mucosa was performed. On days 0 (negative controls), 8 and 10 (other groups), parotid glands were collected. To evaluate oxidative biochemistry, the following analyses were performed: Antioxidant capacity test against peroxyl radicals (ACAP), Lipid Peroxidation Assay (LPO), and Nitric oxide metabolite (NOx). Additionally, histopathological, histomorphometric, and histochemical analyses were performed. Statistical analysis was performed using two‐way ANOVA and Tukey's post‐test (*p* < 0.05).

**Results:**

Clarified açaí, alone or associated with photobiomodulation, increased antioxidant levels compared to the positive control on days 8 and 10. Regarding lipid peroxidation and nitric oxide metabolite, the positive control showed higher levels than the other groups. The morphological assessment showed that the clarified açaí and photobiomodulation groups maintained similar structures of the parenchyma, stroma, and acini to the negative control.

**Conclusions:**

The study results demonstrated that clarified açaí and photobiomodulation conferred biochemical and structural protection to the parotid glands against chemotherapy–induced damage.

## 1 Introduction

Certain medical conditions and medications can affect the salivary glands, decreasing their function and altering the flow and composition of saliva. Chemotherapeutics are among these drugs, inducing hypofunction of the salivary glands, which can cause or aggravate several deleterious consequences in the oral cavity, such as oral mucositis (OM), directly affecting the quality of life of patients undergoing anticancer treatment [1]. Therapeutic strategies have been used to promote comfort and improve the adverse effects of salivary gland hypofunction and chemotherapy‐induced xerostomia; however, there is no strong evidence of the effectiveness of some of these therapies, which limits their use [1, 2].

Photobiomodulation (PBM) has been widely investigated for its therapeutic potential using low‐intensity light sources, such as lasers, light‐emitting diodes (LEDs), and broadband light in the visible and infrared spectra, with the purpose of biostimulating or bioinhibiting the metabolic activity of tissues [3, 4]. PBM can be local, promoting healing and tissue repair, analgesia, and modulation of inflammation; however, systemic effects of local PBM irradiation have also been reported [3]. Therefore, split‐mouth studies are not recommended when using PBM [5, 6]. Although the use of PBM has already been established and recommended for the prevention and treatment of some oral diseases, there is still limited scientific evidence in cases of salivary gland dysfunction and xerostomia [1], especially after use for OM without specific irradiation of the gland.

Moreover, some studies have explored natural products as adjuvants to conventional chemotherapy and radiotherapy treatments [2, 7, 8]. These products have aroused interest due to their easy access, lower cost, and low or no toxicity compared to traditional treatments, in addition to their beneficial bioactive properties [8]. *Euterpe oleracea* Mart. (açaí), widely consumed in the Amazon, exhibits pharmacological, anti‐inflammatory, antioxidant, and anticancer properties. Being rich in bioactive phenolic compounds, including anthocyanins, which are flavonoid pigments that range from red to violet, açaí is recognized for its potential therapeutic applications ([9]). There are no reports in the literature on the effects of açaí specifically on the parotid glands in chemotherapy treatment regimens.

Thus, some questions arise: Can photobiomodulation impact the oxidative metabolism and morphology of salivary glands without direct irradiation? Could the known antioxidant properties of açai modulate these effects? Therefore, the present study aimed to investigate, in a model of induced OM, the biochemical and morphological effects of the application of photobiomodulation in the oral mucosa and supplementation with clarified açaí, used alone and in combination, in the parotid salivary glands of rats.

## 2 Materials and Methods

### 2.1 Experimental Groups

**FIGURE 1:**
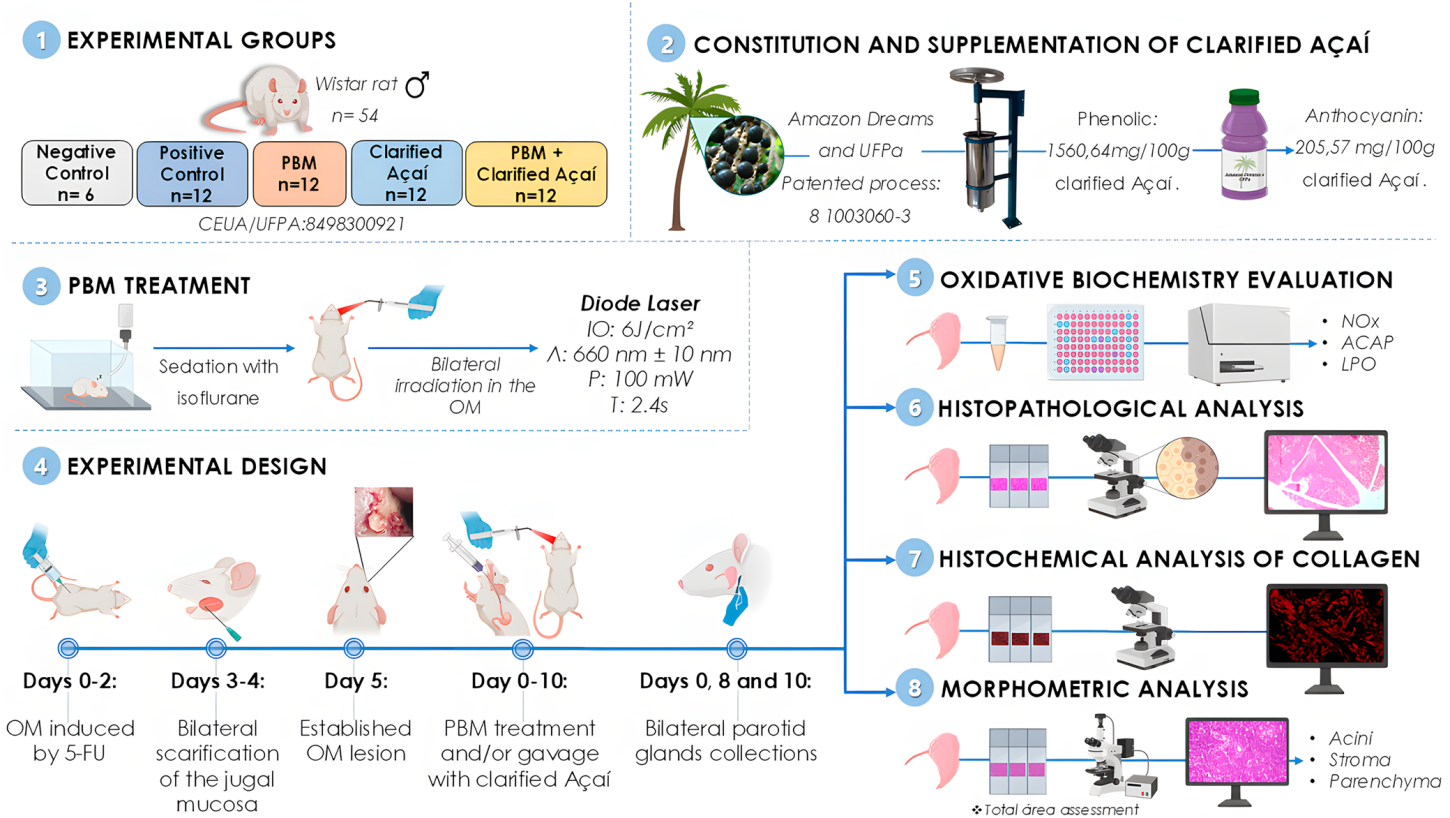
Methodological summary of chemical induction of oral mucositis. Collection of parotid glands, and analyses. (1) Experimental groups, (2) Constitution and supplementation of clarified açaí, (3) Photobiomodulation treatment, (4) Experimental design, (5) Oxidative Biochemistry evaluation, (6) Histopathological analysis, (7) Histochemical analysis of collagen fibers, (8) Morphometric analysis.

This study adhered to the ARRIVE guidelines for animal research (National Research Council) [10], as shown in Figure 1. The experimental procedures were approved by the Ethics Committee for Experimental Animals of the Federal University of Pará (UFPA) (project no. 8498300921), following the recommendations of the NIH Guide for the Care and Use of Laboratory Animals. The sample size calculation was based on Thieme et al. [4], for five groups. Wistar rats (*n* = 54; *Rattus norvegicus* ), aged 90 days, were kept under controlled conditions (temperature: 25°C ± 1°C, humidity: 40%–70%, 12/12 h light/dark cycle).

All rats were obtained from the UFPA Central Animal Facilities. The animals were fed a standard rodent diet with water provided ad libitum, acclimatized at the Faculty of Pharmacy and Biochemistry's Experimental Animal Facility, and assigned to the following groups:

- Negative control (*n* = 6): Standard conditions, no mucositis induction;
- Positive control (*n* = 12): Mucositis was induced, no treatment, and only distilled water was administered by gavage;
- PBM (*n* = 12): Mucositis was induced and PBM was applied locally to OM;
- Clarified Açaí (*n* = 12): Mucositis was induced and treated with clarified açaí via gavage.
- PBM + Clarified Açaí (*n* = 12): Mucositis was induced and treated with local PBM and clarified açaí.

For the collection of parotid glands, on experimental Day 0, only six animals from the negative control group were euthanized; then, on days 8 and 10, eight animals per group were randomly euthanized for analysis.

### 2.2 The Experimental Model of Oral Mucositis

OM was induced according to the protocol proposed by [11] and adapted from [12]. Except for the negative control, all animals in the other groups received an intraperitoneal injection of 5‐fluorouracil (5‐FU) (Fluoro‐Uracil 500 mg/mL, Sigma‐Aldrich, USA) at a dose of 60 mg/kg on Day 0 and 40 mg/kg on Day 2, followed by bilateral scarification of the buccal mucosa on days 3 and 4. On Day 5, all animals were evaluated for the clinical identification of OM.

### 2.3 Photobiomodulation Parameters and Application

**FIGURE 2:**
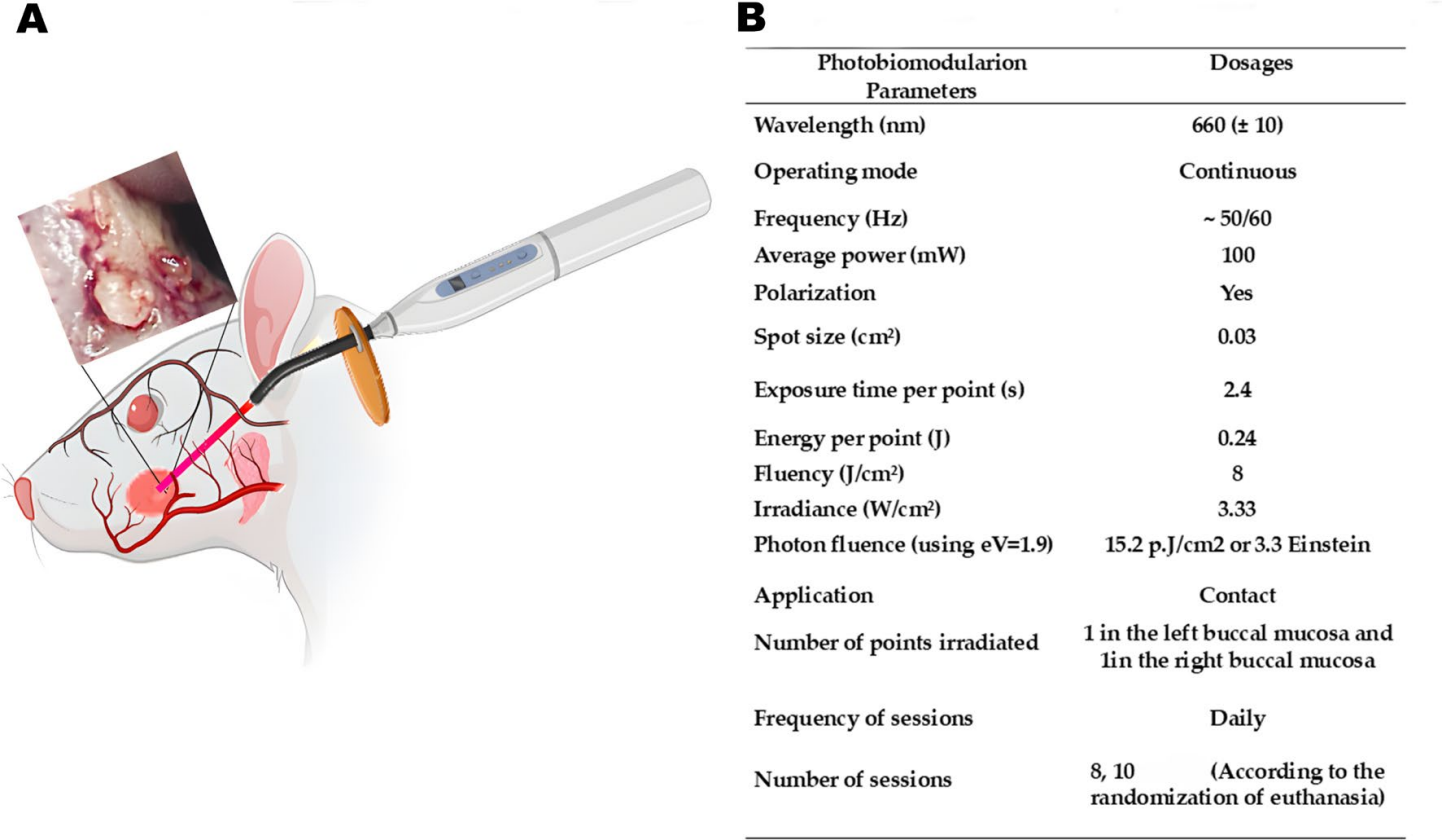
Photobiomodulation treatment. (A): Methodological summary of treatment (B): Laser parameters used in photobiomodulation treatment.

Daily, before use, the output power of the laser was checked with a power meter (LaserCheck, MMOptics Ltda, São Carlos, Brazil). Consecutively, from Day 0 to Day 10, the animals received photobiomodulation therapy. A diode laser (InGaAIP) (MMOptics Ltda, São Carlos, Brazil) emitting red light at a wavelength of 660 nm (±10 nm) was utilized. Figure 2 presents the parameters used, based on the study by Thieme et al. [4], which included the intraoral application mode as well as perpendicular to a central point of the OM lesion, in the mucosa of the right and left sides. Since our objective was to investigate the possible secondary or systemic effects of photobiomodulation, no direct irradiation was performed on the parotid glands.

### 2.4 Constitution of Clarified Açaí

Clarified açaí was prepared following a patented process (PI 1003060‐3, August 4, 2010). The juice obtained was microfiltered and clarified, resulting in a thin, translucent, burgundy‐colored liquid. This juice does not contain lipids, proteins, or fibers, but rather high concentrations of phenolic compounds. The composition of total phenolic compounds and anthocyanins was quantified using HPLC‐DAD, as described by Dos Santos et al. [9]. The analysis revealed 3143.12 mg Eq. gallic acid/L of phenolic compounds, including 112.20 mg/L cyanidin‐3‐glucoside, 543.30 mg/L cyanidin‐3‐rutinoside, 184.15 mg/L homoorientin, 144.81 mg/L orientin, 13.06 mg/L deoxyhexose taxifolin, 10.57 mg/L vitexin, and 10.18 mg/L isovitexin. From Day 0 to Day 14, the Clarified Açaí and PBM + Clarified Açaí groups received clarified açaí supplementation via gavage (1 mL/100 g body weight).

### 2.5 Parotid Gland Collection Procedures

Six animals from each group were randomly anesthetized with ketamine hydrochloride (90 mg/kg) and xylazine hydrochloride (10 mg/kg). After loss of reflexes, the rats were euthanized, and the pair of parotid glands was collected for the analysis.

### 2.6 Oxidative Biochemistry Evaluation

Immediately after collection, the parotid glands were washed in saline solution, subject to freezing with liquid nitrogen, and stored at −80°C. For the analysis, the samples were resuspended in 20 mM Tris–HCl, pH 7.4, at 4°C, using sonic disaggregation (approximate concentration of 1 g/mL). The lysate was stored at −80°C until processing. Quantification of proteins in the samples was performed following the Bradford method [13] and reported as a percentage of the control.

#### 2.6.1 Antioxidant Capacity Against Peroxyl Radicals Test (ACAP)

This analysis follows a protocol proposed by [14], which measures antioxidant capacity in response to reactive oxygen species (ROS) added to each collected parotid gland sample. Samples were prepared by homogenization (1:5 w/v) in Tris–HCl (20 mM, pH 7.4). The homogenates were centrifuged at 10000 × *g* for 20 min at 4°C, and the resulting supernatants were separated for analysis. Afterward, reaction buffer (127.5 μL) containing 30 mM HEPES (pH 7.2), 200 mM KCl, and 1 mM MgCl2 was added to the sample wells. In a microplate, the samples were organized in triplicate, and each well received 7.5 μL of 2,2′‐azobis 2‐methylpropionamidine dihydrochloride (ABAP; 4 mM; Sigma‐Aldrich, St. Louis, MI, USA) for peroxyl radical generation at 35°C. In another microplate, the samples received the same volume of ultrapure water instead of ABAP.

To quantify ROS, 10 μL of 2′,7′‐dichlorofluorescein diacetate (H2DCF‐DA, Invitrogen, Waltham, MA, USA) was added at a final concentration of 40 nM. The thermal decomposition of ABAP and ROS formation was monitored for 60 min, with readings every 5 min on a fluorescence microplate reader using the fluorescence method (Victor X3; Perkin Elmer, Waltham, MA, USA). Total fluorescence was quantified by integrating the fluorescence units (FU) over the data acquisition interval, after fitting the experimental curves to a second‐order polynomial function. The results were expressed as the area difference (FU × min) between the conditions with and without the addition of ABAP, using the area corresponding to basal ROS generation, in the absence of ABAP, as the reference value (background). The relative difference between the areas was used as a parameter to estimate the antioxidant capacity of the sample. The results were expressed as the inverse of the relative area and as a percentage of the control.

#### 2.6.2 Lipid Peroxidation Assay (LPO)

The level of lipid peroxidation was determined using the method proposed by Esterbauer and Cheeseman [15], based on the reaction of polyunsaturated fatty acid metabolites, malonaldehyde (MDA) and 4‐hydroxyalkenes (4‐HA), with N‐methyl‐2‐phenylindole 10.3 mM (NMFI). This reaction, in the presence of methanesulfonic acid, produces a stable chromophore, whose color is proportional to the concentration of oxidized lipids and can be measured spectrophotometrically. For this purpose, the lysates were centrifuged at 3512 × *g* for 10 min at 4°C, and the supernatant was separated into aliquots for determination of lipid peroxidation and protein concentration. To 260 μL of NMFI diluted in methanol (1:3) and 60 μL of methanesulfonic acid, 80 μL of the MDA standard solutions or samples were added. Then, this mixture was heated at 45°C for 40 min. After this period, the reading was performed (*λ* = 570 ηm), and the results were expressed in nanomoles per microgram (nmol/μg) of protein.

#### 2.6.3 Nitric Oxide Metabolite (NOx)

For this determination, the lysate was thawed and centrifuged at 14,000 rpm for 10 min at 4°C. The supernatant was separated into aliquots for determination of nitrite and protein concentration. The nitrite concentration was determined based on the reaction of these with the Griess reagent (0.1% naphthylethylenediamine and 1% sulfanilamide in 5% phosphoric acid—1:1). Fifty microliters of the supernatant or standard nitrite solutions were added to 100 μL of the Griess reagent and incubated for 20 min at room temperature [16]. The results were expressed in micromoles per microgram (μmol/μg) of protein.

### 2.7 Histological Analyses

The samples were immersed in 10% formalin for 48 h for fixation. Subsequently, the glands were processed by being dehydrated in increasing solutions of ethanol (70%, 80%, 90%, absolute 1, and absolute 2), diaphonized in xylene, and then embedded in paraffin. The samples were then re‐soaked in paraffin for 30 min and embedded using Leuckart squares. The blocks were cut into sagittal sections using a manual microtome (YD‐315 Rotary Microtome, YIDI Medical Appliance Factory), adjusted to 5 μm thick sections, and stained with Hematoxylin and Eosin for histopathology and morphometry.

#### 2.7.1 Histopathological and Morphometric Analyses

Two blinded pathologists independently analyzed the slides qualitatively using a microscope (Eclipse E200, Nikon, Tokyo, Japan; magnification, ×40). The Cohen's kappa statistic was applied to assess interobserver agreement, resulting in excellent agreement (*κ* = 0.93). Secretory units, the ductal system, and surrounding stroma were examined to identify potential pathological alterations in the glandular tissue, lining epithelium, and connective tissue of the parotid gland. A digital camera (Eclipse Ci‐S, Nikon, Tokyo, Japan) coupled to a microscope (Eclipse Ci‐S, Nikon, Tokyo, Japan) with a 40× objective was used to record images at a resolution of 2880×2048, with an optical magnification of ~40× and an apparent magnification of ~760× for each sample. Four random sagittal sections of the glands were evaluated, with an average of four fields per section. Using digital image analyzer software ImageJ (NIMH, NIH, Bethesda, MD, USA, http://rsbweb.nih.gov/ij/), with a color threshold present in Gabriel Landini's morphological analysis set plugin [17], morphometric evaluations of the tissues were made, expressed in μm^2^, including total acini areas, total parenchyma, and total stroma ([18]).

#### 2.7.2 Histochemistry Analysis

To evaluate the collagen content of the parotid salivary glands, the samples were subjected to a battery of xylene and alcohol solutions (absolute, 90%, 80%, and 70%), stained with PicroSirius Red [19], and counterstained with Harris hematoxylin. Three sections of each sample were then examined under a polarized light microscope (Eclipse Ci‐S, Nikon, Tokyo, Japan, 40× magnification), and three photomicrographs of each region were obtained using a color digital camera (Eclipse Ci‐S, Nikon, Tokyo, Japan; 40× magnification) attached to the microscope. The threshold tool in ImageJ software (NIMH, NIH, Bethesda, MD, USA, http://rsbweb.nih.gov/ij/) was used to delineate the collagen area, using the arithmetic mean of the sections to determine the collagen area of the sample, which was measured in μm^2^.

### 2.8 Statistical Analysis

Statistical analysis was performed using GraphPad Prism (version 8.0; San Diego, CA, USA). The data were submitted to the Shapiro–Wilk test, which indicated normal distribution (*p* > 0.05), after which two‐way ANOVA with Tukey's post hoc test was used, assuming a statistical significance value of *p* < 0.05. Results are expressed as mean ± standard error.

## 3 Results

### 3.1 Oxidative Biochemistry Evaluation

**FIGURE 3:**
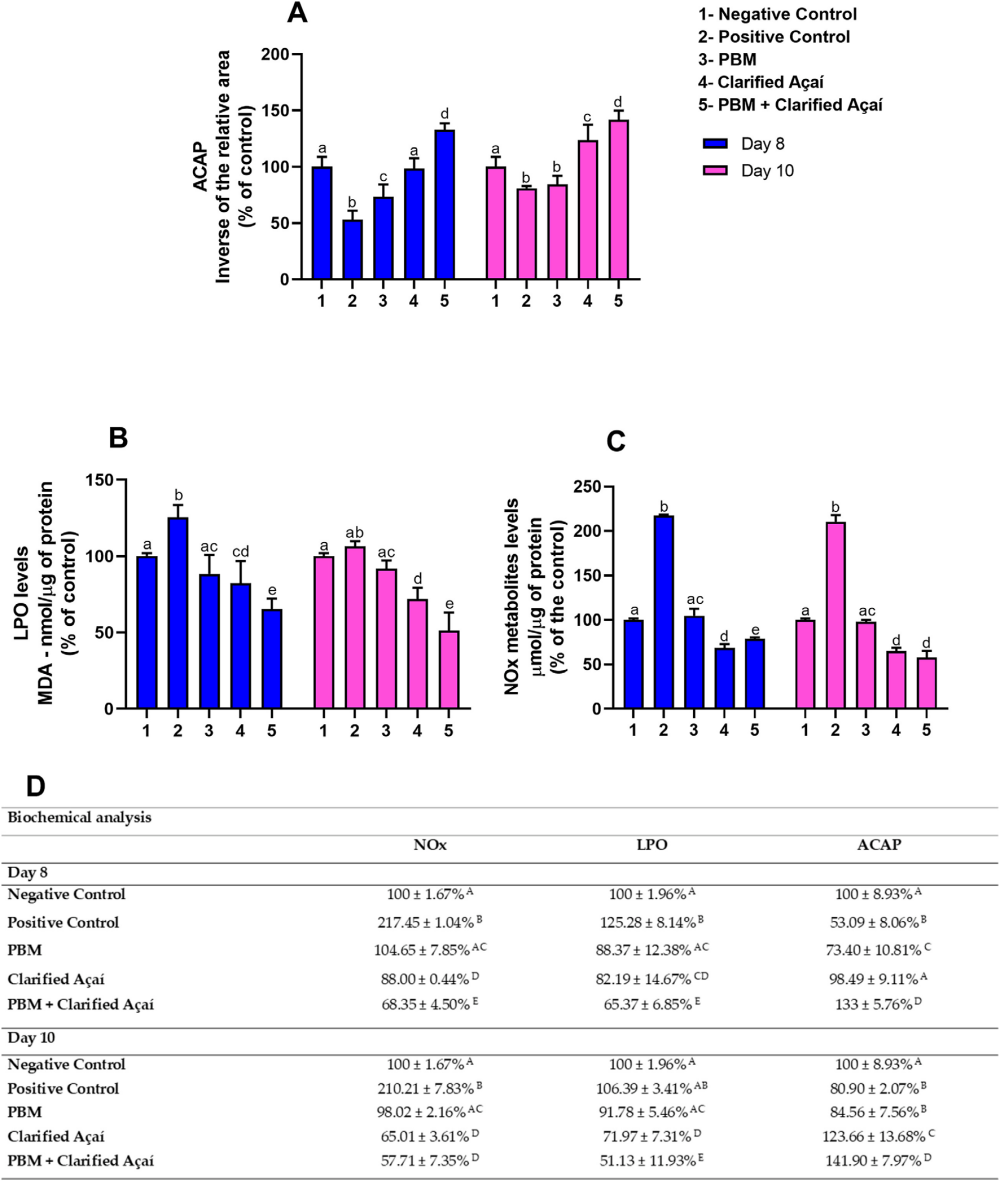
Effects of Photobiomodulation and Clarified Açaí on the oxidative biochemistry of the parotid gland. (A): Antioxidant capacity test against peroxyl radicals (ACAP). (B): Lipid Peroxidation Assay (LPO). (C): Nitric oxide (NOx) metabolites. (D): Quantitative results expressed as mean ± standard error of the mean. Two‐way ANOVA for parametric data and Tukey's post hoc test; letters represent the statistical significance of the differences (*p* < 0.05).

Clarified açaí administered alone or associated with PBM significantly increased antioxidant capacity levels compared to the positive control on days 8 and 10 (Day 8—positive control: 53.09 ± 8.06; Clarified Açaí: 98.49 ± 9.11; *p* < 0.0001; PBM: 73.40 ± 10.81; *p* = 0.0018; PBM + Clarified Açaí: 133 ± 5.76; *p* < 0.0001; Day 10—positive control: 80.90 ± 2.07; Clarified Açaí: 123.66 ± 13.68; *p* < 0.0001; PBM + Clarified Açaí: 141.90 ± 7.97; *p* < 0.0001), as shown in Figure 3A. It is important to highlight that the results showed a large effect size (partial *η* ^2^ = 0.743).

For the lipid peroxidation analysis, the PBM, Clarified Açaí, and PBM + Clarified Açaí groups showed significantly lower levels compared to the positive control on days 8 and 10 (Day 8—positive control: 125.28 ± 8.14; Clarified Açaí: 82.19 ± 14.67; *p* < 0.0001; PBM: 88.37 ± 12.38; *p* < 0.0001; PBM + Clarified Açaí: 65.37 ± 6.85; *p* < 0.0001; Day 10—positive control: 106.39 ± 3.41; Clarified Açaí: 71.97 ± 7.31; *p* < 0.0001; PBM: 91.78 ± 5.46; *p* = 0.0355; PBM + Clarified Açaí: 51.13 ± 11.93; *p* < 0.0001). The results showed a large effect size (partial *η* ^2^ = 0.562) (Figure 3B).

On all evaluated days, the positive control exhibited high levels of nitric oxide metabolites, while all experimental groups maintained low levels, with significant differences and a very large effect size (Positive control: Day 8–217.45 ± 1.04; *p* < 0.0001; Day 10–210.21 ± 7.83; *p* < 0.0001; partial *η* ^2^ = 0.879) (Figure 3C).

### 3.2 Histological Analysis

**FIGURE 4:**
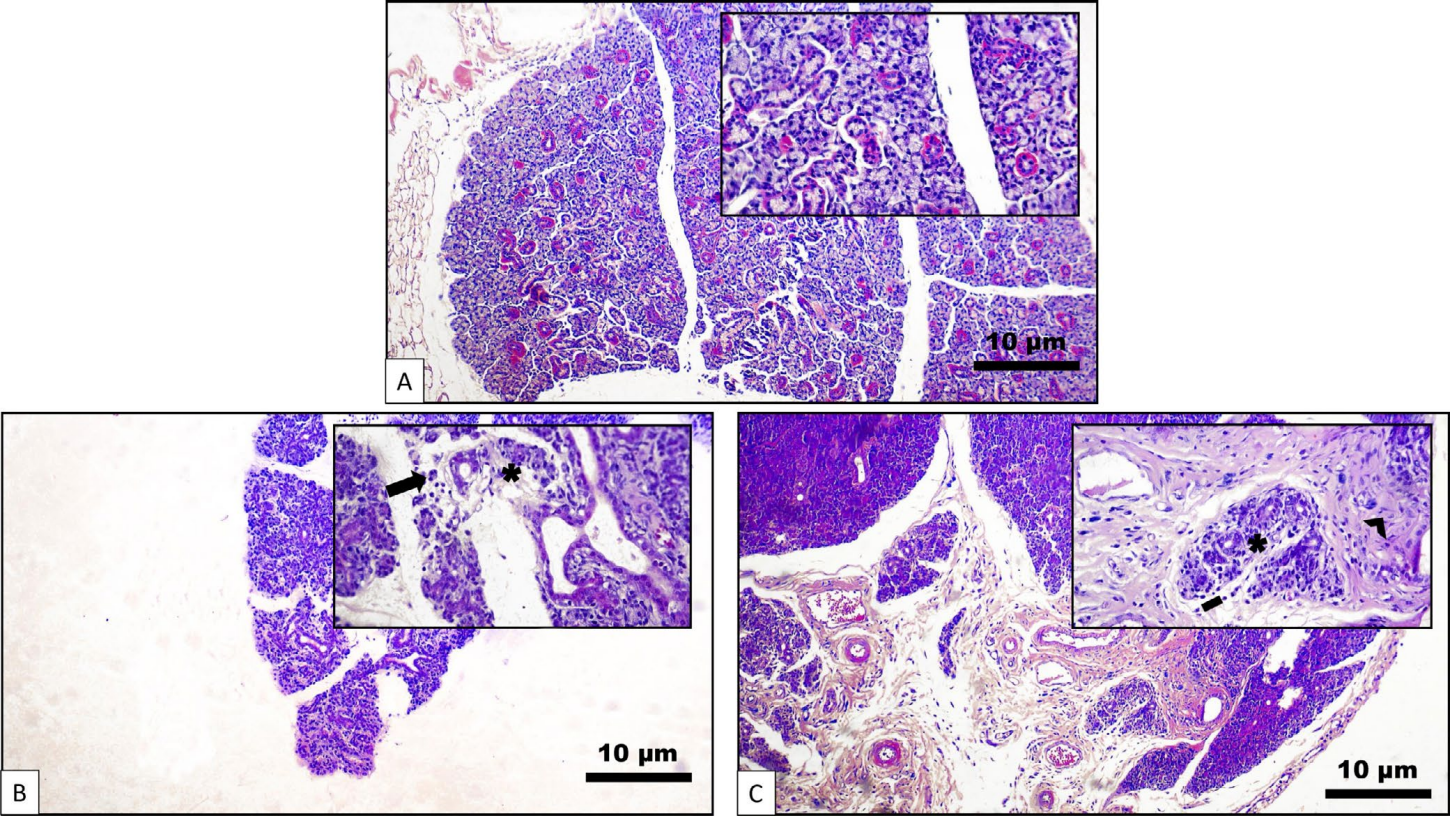
Morphological changes evidenced in the parotid gland exposed to 5‐FU between 8 and 10 days. (A) Parotid control group at Day 0 demonstrating normal glandular parenchyma (H&E, 100×, inset: 400×). (B) Analysis at 8 days. Discrete mononuclear inflammatory infiltrate in the stroma and acinar atrophy (H&E, 100×, inset: 400×). (C) Analysis at 10 days. Mild mononuclear inflammatory infiltrate, interstitial fibrosis, and acinar atrophy (H&E, 100×, inset: 400×). Scale bar: 10 μm.

Based on comparisons with the negative control group, which presented normal glandular parenchyma, on Day 8 the positive control group presented moderate mononuclear inflammatory infiltrate and the presence of mast cells in the stroma, in addition to chronic sialadenitis, acinar atrophy, ductal ectasia, interstitial edema, many vacuoles, and fibrosis. On Day 10, the same group showed moderate mononuclear infiltration, mild interstitial fibrosis, focal acinar atrophy, duct ectasia, interstitial edema, and mast cells in the stroma (Figure 4).

**FIGURE 5:**
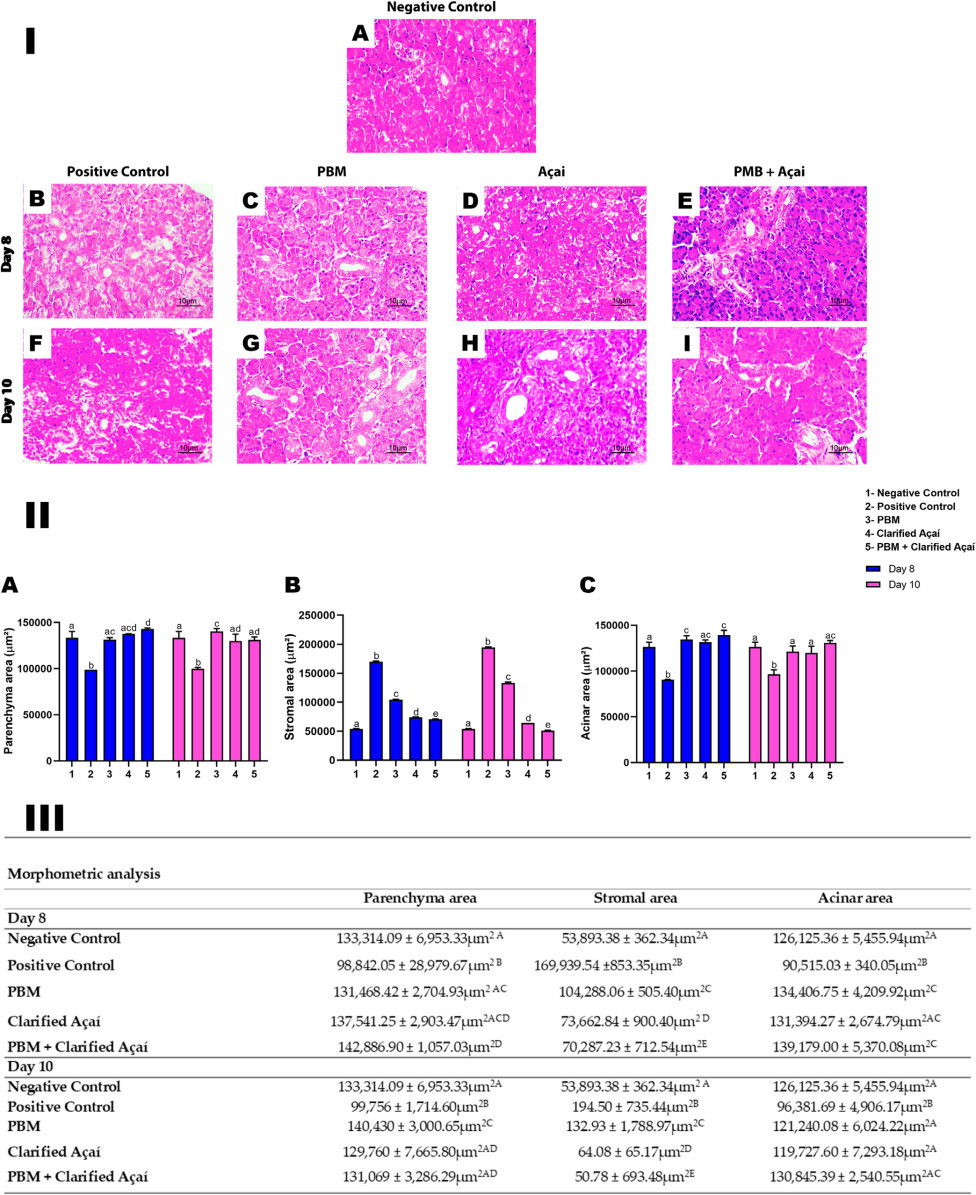
Effect of Photobiomodulation and Clarified Açaí on morphometric analyses of the parotid gland at 8 and 10 days. I—Photomicrographs representing total areas of parenchyma (μm^2^), stroma (μm^2^), and acini (μm^2^) of the gland (H&E, 400×). (A) Negative Control. Intervention groups at 8 days: (B) Positive Control, (C) PBM, (D) Clarified Açaí, (E) PBM + Clarified Açaí (H&E, 400×). Intervention groups at 10 days: (F) Positive Control, (G) PBM, (H) Clarified Açaí, (I) PBM + Clarified Açaí (H&E, 400×). Intervention groups at 14 days: (J) Positive Control, (K) PBM, (L) Clarified Açaí, (M) PBM + Clarified Açaí (H&E, 400×). Scale bar: 10 μm. II—Graphs of statistical analyses between parenchyma, stroma, and acini variables. (A): Total area (μm^2^) of parotid parenchyma (B): Total area (μm^2^) of parotid stroma (C): Total area (μm^2^) of parotid acini. III—Quantitative representation of morphometric analysis using two‐way ANOVA for parametric data and Tukey's post hoc test; letters represent significance of statistical differences (*p* < 0.05).

Clarified açaí and photobiomodulation, when used alone or associated, maintained the morphological structures of the parenchyma, stroma, and acini, presenting similar values to the negative control on Day 8. In addition, all experimental groups showed better results than the positive control group, which showed a reduction in parenchymal structure, an increase in stroma, and a reduction in acini. Conversely, all groups, except the positive control, presented similar parotid salivary gland morphology to the negative control and a large effect size for parenchyma (partial *η* ^2^ = 0.569), very large for stroma (partial *η* ^2^ = 0.996), and large for acini (partial *η* ^2^ = 0.620) (Figure 5).

**FIGURE 6:**
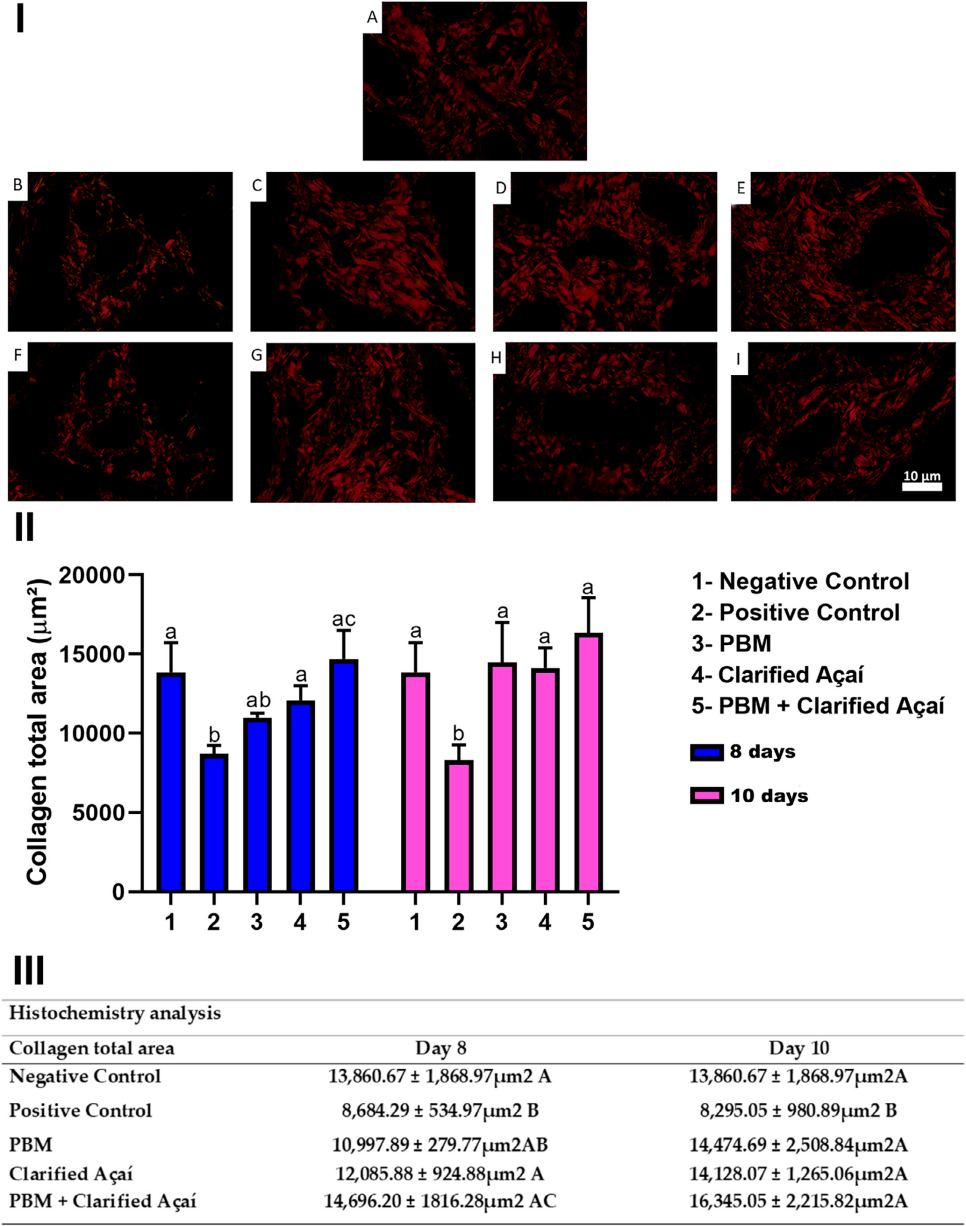
Effect of Photobiomodulation and Clarified Açaí on histochemical analysis of parotid gland collagen fibers at 8 and 10 days. I—Photomicrographs represent parotid gland collagen fibers (H&E, 400×). (A) Negative Control. Intervention groups at 8 days: (B) Positive Control, (C) PBM, (D) Clarified Açaí, (E) PBM + Clarified Açaí (H&E, 400×). Intervention groups at 10 days: (F) Positive Control, (G) PBM, (H) Clarified Açaí, (I) PBM + Clarified Açaí (H&E, 400×). Scale bar: 10 μm. II—Graph of statistical analysis between collagen fiber area (μm^2^). III—Quantitative representation of the analysis using two‐way ANOVA for parametric data and Tukey's post hoc test. Letters represent significance of statistical differences (*p* < 0.05).

On days 8 and 10, the positive control exhibited a reduction in collagen area compared to the negative control (Negative control: 13860.67 ± 1868.97; Positive control: Day 8–8684.29 ± 534.97; *p* < 0.0001; Day 10–8295.05 ± 980.89; *p* < 0.0001). On Day 8, no significant difference was observed between the negative control and PBM‐treated groups (10997.89 ± 279.77; *p* = 0.0510). However, the Clarified Açaí and PBM + Clarified Açaí groups showed a significant increase in collagen area compared to the positive control (Clarified Açaí: 12085.88 ± 924.88; *p* = 0.0132; PBM + Clarified Açaí: 14696.23 ± 1816.28; *p* < 0.0001). Notably, the PBM + Clarified Açaí group exhibited a significant increase in collagen compared to the PBM group (*p* = 0.0059) (Figure 6).

On Day 10, all experimental groups showed a significant increase in collagen fiber area compared to the positive control, with a small/moderate effect size (partial *η* ^2^ = 0.137); (PBM: Day 10–14474.69 ± 2508.84; *p* < 0.0001; Clarified Açaí: Day 10–14128.07 ± 1265.06; *p* < 0.0001; PBM + Clarified Açaí: Day 10–16345.05 ± 2215.82; *p* < 0.0001).

## 4 Discussion

The findings of the current study demonstrated that clarified açaí, whether used alone or in combination with photobiomodulation, resulted in enhancement in the antioxidant pathway and a reduction in lipid peroxidation levels and nitric oxide metabolites in the parotid gland. This indicates a protective effect against oxidative biochemical damage. Additionally, the histopathological analysis revealed that groups treated with clarified açaí exhibited less structural damage.

The mechanism by which 5‐FU affects protein synthesis in the rat parotid gland may involve its incorporation into cellular RNA [20], leading to oxidative stress, inflammation, and damage to vital structures that can cause cell apoptosis and compromise salivary gland function [1]. A previous study showed that 5‐FU exposure increases lipid peroxidation in the parotid gland due to excessive ROS release, reducing antioxidant enzymes, such as superoxide dismutase and reduced glutathione [21]. This inflammatory reaction leads to a decrease in functional cells, glandular atrophy, and loss of salivary flow, potentially causing irreversible damage to acini and ducts and resulting in hyposalivation and xerostomia [20, 21].

Various therapeutic strategies have been analyzed for both the prevention and alleviation of adverse effects related to salivary gland dysfunction induced by chemotherapy or chemoradiotherapy. Among the preventive measures, intensity‐modulated radiotherapy stands out as the most consistent intervention for reducing the prevalence of xerostomia. However, results regarding the objective preservation of salivary function are heterogeneous, with some studies reporting no significant improvement in salivary flow rates. Furthermore, the widespread implementation of this technique may be limited by factors such as technological availability and high cost. Another preventive approach under investigation is acupuncture, which has shown potential in reducing dry mouth. However, the available data present significant limitations, such as variability in application protocols and a scarcity of comparative studies with other preventive interventions, which hinder the consolidation of this strategy as a recommended clinical practice [22].

Regarding the management of existing symptoms, strategies such as the use of sugar‐free gum or candies have been widely used for symptomatic relief. Although they provide temporary oral lubrication, these interventions do not impact the functional recovery of the salivary glands, and their efficacy may vary depending on the formulation. Furthermore, in patients with sensitized mucosa or OM, these products may be poorly tolerated. The use of sialagogues, such as pilocarpine and cevimeline, represents another option for managing hyposalivation. These drugs stimulate the remaining glandular parenchyma and can provide temporary symptomatic relief. However, the adverse effects associated with these drugs, such as excessive sweating, headache, gastrointestinal discomfort, and increased urinary frequency, limit their adherence in certain patient groups. Other approaches have also been studied, such as transcutaneous electrical stimulation, but there is still a lack of robust evidence on their efficacy. The results observed to date are mostly subjective, with improvement limited to xerostomia symptoms and no evidence of a sustained increase in salivary flow [1].

In the present study, we investigated another strategy: photobiomodulation therapy. Although studies have been conducted specifically on salivary glands, there is still insufficient scientific evidence to recommend or contraindicate the use of photobiomodulation therapy in the salivary glands of patients undergoing antineoplastic therapies [1]. Our objective was to analyze whether photobiomodulation, even when applied at a distance from the salivary glands of rats, could trigger secondary or systemic biochemical and morphological effects. To this end, we used a photobiomodulation protocol that had already shown positive results in a previous study on OM ([4]). Furthermore, laser irradiation began on Day 0 of chemical induction, following clinical recommendations for the prevention and treatment of damage caused by chemotherapy [8].

The results showed that the positive control group presented significantly lower levels of antioxidant capacity on days 8 and 10, accompanied by an increase in nitric oxide metabolite levels, reflecting the negative impact of the chemotherapy agent on redox balance. In contrast, the groups treated with clarified açaí and/or photobiomodulation maintained high antioxidant levels on days 8 and 10. Furthermore, lower levels of lipid peroxidation were observed on days 8 and 10, suggesting a rapid and effective antioxidant response. Notably, photobiomodulation, both alone and in combination with açaí, was able to preserve nitric oxide metabolite values at levels similar to those of the negative control group, which did not receive chemotherapy.

Photobiomodulation is a non‐thermal and painless process that results in the activation of endogenous chromophores and triggers biochemical and biophysical events, which can be both biostimulatory and bioinhibitory. The effects of this therapy have been reported locoregionally, but also at distant sites and systemically [23, 24]. Studies evaluating the effects of photobiomodulation applied locally to the salivary glands have highlighted the following: ductal and epithelial growth, increased protein synthesis, reduced cell death markers, decreased inflammation, changes in calcium levels, increased mitochondrial activity, increased ATP availability, increased cell proliferation, improved blood circulation, and changes in the flow and chemical composition of saliva [25, 26, 27].

Some studies have reported the systemic effects of photobiomodulation therapy [25, 26, 28]. These effects were investigated in the salivary glands of a patient with drug‐induced xerostomia [29]; however, no studies were found that specifically address patients undergoing chemotherapy. Thus, our results showed that photobiomodulation maintained similar pro‐oxidant levels and glandular morphometry to those of the negative control group. The mechanisms by which photobiomodulation acted in this case are still unclear and require further investigation. However, based on current knowledge, we suggest that, since the parotids were not directly irradiated, the effects obtained were due to transmucosal irradiation of an intensely vascularized region in the rats. In addition, if this indeed occurred, the action of the therapy occurred in the blood. This mode of action has been proposed for Vascular Photobiomodulation (VPBM), which is applied to areas with a high concentration of blood vessels, such as the transnasal region or the floor of the mouth (sublingual). VPBM is a modification of a technique proposed around 40 years ago, Intravascular Laser Irradiation of Blood (ILIB), which works by stimulating the enzyme superoxide dismutase, essential for breaking down the mechanism of formation of free radicals generated by oxygen and protecting against the reactivity and toxicity of these radicals. Thus, antioxidant effects are stimulated, inflammation is modulated, and the hemorheological capacity of blood cells is improved [25, 26]. However, we reiterate that further studies are needed to confirm or refute this hypothesis. On the other hand, several studies demonstrate that the bioactive compounds in açaí, especially anthocyanins and flavonoids, have high antioxidant activity and are rapidly absorbed by the gastrointestinal tract after oral administration, with systemic distribution, and promote effects in various tissues, such as the liver, intestinal mucosa, oral mucosa, and alveolar bone [9, 30, 31].

The pharmacodynamics of açaí are primarily related to the modulation of oxidative stress, with increased antioxidant defenses, such as reduced glutathione and superoxide dismutase, and reduced markers of lipid peroxidation, in addition to anti‐inflammatory and tissue regeneration effects ([30, 32]). These effects have already been demonstrated in the intestinal mucosa of animals with 5‐FU‐induced mucositis ([32]), as well as in the oral mucosa of rats with chemically treated and induced wounds, in which açaí accelerated epithelial repair ([33]). In our study, the animals received açaí from the first day of chemical induction with 5‐Fu, at a dose of 1 mL/100 g body weight. This dosage used the allometric calculation exemplified in a previous study [34]. The açaí antioxidant dose used in humans should be converted to Wistar rats, applying the Km correction factor, which relates to the weight and body surface area of each species. According to the proposed values, the Km for adult humans is 37, while for rats it is 6. Therefore, the equivalent dose in rats is obtained by multiplying the human dose (in mg/kg) by the quotient between the factors (37/6). This methodology is considered the most appropriate because it better correlates relevant physiological parameters, such as basal metabolism, blood volume, and renal function, ensuring greater reliability in extrapolating doses between humans and animal models, as in the case of investigating the antioxidant effects of açaí [34].

Although available studies do not present specific data on the pharmacokinetic parameters of açaí, such as peak plasma time, bioavailability, or half‐life after oral administration, biological effects were consistently observed in animal models within a relatively short time after the start of treatment. For example, in the study using the intestinal mucositis model, the regenerative effects of açaí pulp were evident as early as the third day after the start of continuous oral administration ([32]). Despite the scarcity of studies targeting salivary glands, it is plausible that the effects observed in other epithelial tissues also extend to these structures. Salivary glands share common pathophysiological mechanisms with the oral and intestinal mucosa, including oxidative stress and inflammation induced by chemotherapy and/or radiotherapy ([33]). Thus, our findings suggest that the combination of photobiomodulation and clarified açaí may have promoted synergistic action. That is, by inducing vasodilation in glandular vessels [3], photobiomodulation may have favored tissue perfusion and, consequently, increased the bioavailability of the bioactive compounds in the açaí. This interaction between the antioxidant mechanisms of açaí and the biostimulatory effects of photobiomodulation may have potentiated the protective and regenerative response in the salivary glands, reducing the deleterious effects of 5‐FU by mitigating the production of ROS and preserving tissue integrity ([32]), as observed in the morphometric analysis.

The morphometric findings in the current study reinforce the maintenance of parenchyma, stroma, and acini structures in the groups that received clarified açaí supplementation. These biochemical findings were associated with the structural alterations found in the morphometric analyses, such as a reduction in total parenchyma levels, followed by an increase in stroma and a reduction in acini. These results are in agreement with previous studies that demonstrated that 5‐FU caused structural alterations in the parotid gland, such as several ruptures in the acini, vesiculation, and shortening of the lamellae of the rough endoplasmic reticulum, in addition to the reduction of transport vesicles and secretory granules, resulting in reduced salivary synthesis and transport [35, 36].

The histopathological findings demonstrated that the untreated group presented acinar atrophy, ductal ectasia, edema, interstitial fibrosis, and an inflammatory process, with the presence of leukocytes and mast cells, which are often responsible for the release of inflammatory mediators, such as cytokines and ROS [33]. In the clarified açaí group, there was mild inflammation on Day 8 and normal glandular parenchyma on Day 10. The isolated PBM resulted in mild inflammation on days 8 and 10. As for the associated group, normal glandular parenchyma was observed in all periods analyzed, thus indicating a protective and anti‐inflammatory effect.

Studies show that oxidative stress can disrupt the extracellular matrix, affecting its components, including collagen, which may undergo altered synthesis, unwinding of its triple helix, or fragmentation of fibronectin and oxidation of fibrinogen [37]. This could explain the histochemical findings showing reduced collagen fibers in the parotid gland after 5‐FU exposure. Supporting this, in vitro studies demonstrate that 5‐FU inhibits fibroblast proliferation [38]. However, collagen fibers were maintained in the treatment groups, suggesting that the antioxidant capacity of clarified açaí [9] effectively mitigated the effects of 5‐FU on the extracellular matrix.

It is important to emphasize that our study was conducted in an animal model, which limits the direct extrapolation of the results to human application. Furthermore, other limitations can be highlighted, such as the sample size, which, although defined based on statistical criteria, may have influenced the robustness of the findings, and the use of only a single chemotherapeutic agent for mucositis induction, which does not fully reflect the complexity of multidrug regimens used in clinical practice. For our findings to be applicable in clinical studies, some important points must be considered, such as standardizing the most appropriate dosage for clarified açaí, interindividual variability in antioxidant response, and developing photobiomodulation protocols that allow for the reproducibility of the findings. We believe that our results should be understood as initial data for further investigation since the protective effects observed in Wistar rats may provide relevant information for the investigation of possible molecular targets and understanding the mechanisms of action of both therapies, as well as for the design of new protocols for the prevention and treatment of salivary glands exposed to chemotherapy, especially by indicating that both clarified açaí supplementation and photobiomodulation have the potential to modulate inflammatory and oxidative processes in these glands.

The clinical application of these strategies could present potential advantages, given their non‐invasive nature, low cost compared to other therapies, good patient acceptance, and favorable safety profile, making them promising adjuvants. Finally, we believe that the synergy between nutritional supplementation and photobiomodulation opens up possibilities for exploration in different clinical contexts, and could be used as a preventive and/or therapeutic strategy in specific populations of vulnerable patients, such as those at high risk of hyposalivation, the elderly, children, or individuals undergoing combined chemotherapy and radiotherapy regimens in the head and neck region [22]. These possibilities confirm the need for future well‐designed and structured translational research, with short and long‐term patient follow‐up, capable of identifying whether the findings of our study hold up in humans and, if so, how long these effects would last.

## 5 Conclusion

This study found that supplementation with clarified açaí, alone or in combination with photobiomodulation, protected against oxidative damage, preserving parotid gland structures, and reducing inflammation caused by chemotherapy. Future studies are needed to elucidate the mechanisms of action of the proposed treatments, as well as to evaluate the findings in humans.

## Author Contributions

W.W.P.M., W.A.B.A, V.R.N.d.S., and H.H.N.F. carried out the experimental phases. W.W.P.M., W.A.B.A, L.O.B., P.F.S.M., J.M.P., H.G.F.M., H.R., R.d.A.F., and M.D.M. analyzed the results. R.R.L. and R.D.d.S.‐R. guided and supervised the execution of all experimental steps. W.W.P.M. and R.D.d.S.‐R. wrote the initial version of the manuscript. All authors reviewed and contributed to the final version of this manuscript and agree with the order in which their names are listed in it.

## Conflicts of Interest

The authors declare no conflicts of interest.

## Supporting information


**Table S1:** Parametric results of the biochemical analysis of nitrite metabolite levels (NOx), lipid peroxidation levels (LPO) and antioxidant capacity against peroxyl (ACAP). The results were plotted in percentage of the control. And results of the morphometric analyzes being total parenchymal area, total stromal area and total acinar area (μm^2^), and histochemistry analysis (Collagen total area μm^2^), of the parotid gland of rats submitted to a chemically induced OM model. Results were expressed as mean ± standard error of mean.
**Table S2:** Parametric results of the biochemical analysis of nitrite metabolite levels (NOx), lipid peroxidation levels (LPO) and antioxidant capacity against peroxyl (ACAP). The results were plotted in percentage of the control. And results of the morphometric analyzes being total parenchymal area, total stromal area and total acinar area (μm^2^), and histochemistry analysis (Collagen total area μm^2^), of the parotid gland of rats submitted to a chemically induced OM model. Results were expressed as mean ± standard error of mean.

## Data Availability

The data that supports the findings of this study are available in the Supporting Information of this article.
