# Supplementary material for: Effects of Clarified Açaí Supplementation and Photobiomodulation on the Parotid Glands of Rats Submitted to Chemotherapy
Source: J Oral Pathol Med. 2025 Oct 24;55(1):120–32. doi: 10.1111/jop.70072 (PMC12774578; doi:10.1111/jop.70072)
Supplement: Supplementary file 1 — Table S1: Parametric results of the biochemical analysis of nitrite metabolite levels (NOx), lipid peroxidation levels (LPO) and antioxidant capacity against peroxyl (ACAP). The results were plotted in percentage of the control. And results of the morphometric analyzes being total parenchymal area, total stromal area and total acinar area (μm2), and histochemistry analysis (Collagen total area μm2), of the parotid gland of rats submitted to a chemically induced OM model. Results were expressed as mean ± standard error of mean. Table S2: Parametric results of the biochemical analysis of nitrite metabolite levels (NOx), lipid peroxidation levels (LPO) and antioxidant capacity against peroxyl (ACAP). The results were plotted in percentage of the control. And results of the morphometric analyzes being total parenchymal area, total stromal area and total acinar area (μm2), and histochemistry analysis (Collagen total area μm2), of the parotid gland of rats submitted to a chemically induced OM model. Results were expressed as mean ± standard error of mean. [file JOP-55-120-s001.docx]

| Biochemical analysis | | | |
| --- | --- | --- | --- |
|  | **NOx** | **LPO** | **ACAP** |
| Day 8 |  |  |  |
| Negative Control  Positive Control  PBM | 100 ± 1.67% ^A^  217.45 ± 1.04% ^B^  104.65 ± 7.85% ^AC^ | 100 ± 1.96% ^A^  125.28 ± 8.14% ^B^  88.37 ± 12.38% ^AC^ | 100 ± 8.93% ^A^  53.09 ± 8.06% ^B^  73.40 ± 10.81% ^C^ |
| Clarified Açaí | 88.00 ± 0.44% ^D^ | 82.19 ± 14.67% ^CD^ | 98.49 ± 9.11% ^A^ |
| PBM + Clarified Açaí | 68.35 ± 4.50% ^E^ | 65.37 ± 6.85% ^E^ | 133 ± 5.76% ^D^ |
| Day 10 |  |  |  |
| Negative Control | 100 ± 1.67% ^A^ | 100 ± 1.96% ^A^ | 100 ± 8.93% ^A^ |
| Positive Control | 210.21 ± 7.83% ^B^ | 106.39 ± 3.41% ^AB^ | 80.90 ± 2.07% ^B^ |
| PBM | 98.02 ± 2.16% ^AC^ | 91.78 ± 5.46% ^AC^ | 84.56 ± 7.56% ^B^ |
| Clarified Açaí | 65.01 ± 3.61% ^D^ | 71.97 ± 7.31% ^D^ | 123.66 ± 13.68% ^C^ |
| PBM + Clarified Açaí | 57.71 ± 7.35% ^D^ | 51.13 ± 11.93% ^E^ | 141.90 ± 7.97% ^D^ |
| Morphometric analysis | | | |
|  | **Parenchyma area** | **Stromal area** | **Acinar area** |
| Day 8 |  |  |  |
| Negative Control  Positive Control  PBM | 133,314.09 ± 6,953.33µm^2 A^  98,842.05 ± 28,979.67µm^2 B^  131,468.42 ± 2,704.93µm^2 AC^ | 53,893.38 ± 362.34µm^2 A^  169,939.54 ±853.35µm^2 B^  104,288.06 ± 505.40µm^2 C^ | 126,125.36 ± 5,455.94µm^2 A^  90,515.03 ± 340.05µm^2 B^  134,406.75 ± 4,209.92µm^2 C^ |
| Clarified Açaí | 137,541.25 ± 2,903.47µm^2 ACD^ | 73,662.84 ± 900.40µm^2 D^ | 131,394.27 ± 2,674.79µm^2 AC^ |
| PBM + Clarified Açaí | 142,886.90 ± 1,057.03µm^2D^ | 70,287.23 ± 712.54µm^2 E^ | 139,179.00 ± 5,370.08µm^2 C^ |
| Day 10 |  |  |  |
| Negative Control | 133,314.09 ± 6,953.33µm^2 A^ | 53,893.38 ± 362.34µm^2 A^ | 126,125.36 ± 5,455.94µm^2 A^ |
| Positive Control | 99,756 ± 1,714.60µm^2 B^ | 194.50 ± 735.44µm^2 B^ | 96,381.69 ± 4,906.17µm^2 B^ |
| PBM | 140,430 ± 3,000.65µm^2 C^ | 132.93 ± 1,788.97µm^2 C^ | 121,240.08 ± 6,024.22µm^2 A^ |
| Clarified Açaí | 129,760 ± 7,665.80µm^2 AD^ | 64.08 ± 65.17µm^2 D^ | 119,727.60 ± 7,293.18µm^2 A^ |
| PBM + Clarified Açaí | 131,069 ± 3,286.29µm^2 AD^ | 50.78 ± 693.48µm^2 E^ | 130,845.39 ± 2,540.55µm^2 AC^ |

**Supplementary material**

| Histochemistry analysis |  |  |
| --- | --- | --- |
| Collagen total area | **Day 8** | **Day 10** |
| Negative Control  Positive Control  PBM | 13,860.67 ± 1,868.97µm^2 A^  8,684.29 ± 534.97µm^2 B^  10,997.89 ± 279.77µm^2AB^ | 13,860.67 ± 1,868.97µm^2A^  8,295.05 ± 980.89µm^2 B^  14,474.69 ± 2,508.84µm^2A^ |
| Clarified Açaí | 12,085.88 ± 924.88µm^2 A^ | 14,128.07 ± 1,265.06µm^2A^ |
| PBM + Clarified Açaí | 14,696.20 ± 1816.28µm^2 AC^ | 16,345.05 ± 2,215.82µm^2A^ |

**Supplementary table 1.** Parametric results of the biochemical analysis of nitrite metabolite levels (NOx), lipid peroxidation levels (LPO) and antioxidant capacity against peroxyl (ACAP). The results were plotted in percentage of the control. And results of the morphometric analyzes being total parenchymal area, total stromal area and total acinar area (µm^2^), and histochemistry analysis (Collagen total area µm^2^), of the parotid gland of rats submitted to a chemically induced OM model. Results were expressed as mean ± standard error of mean.

**Supplementary table 2.** Parametric results of the biochemical analysis of nitrite metabolite levels (NOx), lipid peroxidation levels (LPO) and antioxidant capacity against peroxyl (ACAP). The results were plotted in percentage of the control. And results of the morphometric analyzes being total parenchymal area, total stromal area and total acinar area (µm^2^), and histochemistry analysis (Collagen total area µm2), of the parotid gland of rats submitted to a chemically induced OM model. Results were expressed as mean ± standard error of mean.

| **NOx** |  |  |  |  |  |
| --- | --- | --- | --- | --- | --- |
| **Tukey's multiple comparisons test** | **Mean Diff.** | **95,00% CI of diff.** | **Significant?** | **Summary** | **Adjusted P Value** |
|  |  |  |  |  |  |
| **Day 8** |  |  |  |  |  |
| **Negative Control vs. Positive Control** | -117,5 | -125,2 to -109,7 | Yes | **** | <0,0001 |
| **Negative Control vs. PBM** | -4,657 | -12,39 to 3,073 | No | ns | 0,4404 |
| **Negative Control vs. Clarified Açaí** | 31,64 | 23,91 to 39,37 | Yes | **** | <0,0001 |
| **Negative Control vs. PBM + Clarified Açaí** | 21,00 | 13,27 to 28,73 | Yes | **** | <0,0001 |
| **Positive Control vs. PBM** | 112,8 | 105,1 to 120,5 | Yes | **** | <0,0001 |
| **Positive Control vs. Clarified Açaí** | 149,1 | 141,4 to 156,8 | Yes | **** | <0,0001 |
| **Positive Control vs. PBM + Clarified Açaí** | 138,4 | 130,7 to 146,2 | Yes | **** | <0,0001 |
| **PBM vs. Clarified Açaí** | 36,30 | 28,57 to 44,03 | Yes | **** | <0,0001 |
| **PBM vs. PBM + Clarified Açaí** | 25,65 | 17,92 to 33,38 | Yes | **** | <0,0001 |
| **Clarified Açaí vs. PBM + Clarified Açaí** | -10,65 | -18,38 to -2,915 | Yes | ** | 0,0026 |
| **Day 10** |  |  |  |  |  |
| **Negative Control vs. Positive Control** | -110,2 | -117,9 to -102,5 | Yes | **** | <0,0001 |
| **Negative Control vs. PBM** | 1,970 | -5,760 to 9,701 | No | ns | 0,9506 |
| **Negative Control vs. Clarified Açaí** | 34,99 | 27,26 to 42,72 | Yes | **** | <0,0001 |
| **Negative Control vs. PBM + Clarified Açaí** | 42,29 | 34,56 to 50,02 | Yes | **** | <0,0001 |
| **Positive Control vs. PBM** | 112,2 | 104,5 to 119,9 | Yes | **** | <0,0001 |
| **Positive Control vs. Clarified Açaí** | 145,2 | 137,5 to 152,9 | Yes | **** | <0,0001 |
| **Positive Control vs. PBM + Clarified Açaí** | 152,5 | 144,8 to 160,2 | Yes | **** | <0,0001 |
| **PBM vs. Clarified Açaí** | 33,02 | 25,29 to 40,75 | Yes | **** | <0,0001 |
| **PBM vs. PBM + Clarified Açaí** | 40,32 | 32,59 to 48,05 | Yes | **** | <0,0001 |
| **Clarified Açaí vs. PBM + Clarified Açaí** | 7,300 | -0,4301 to 15,03 | No | ns | 0,0727 |
| **LPO** |  |  |  |  |  |
| **Tukey's multiple comparisons test** | **Mean Diff.** | **95,00% CI of diff.** | **Significant?** | **Summary** | **Adjusted P Value** |
| **Day 8** |  |  |  |  |  |
| **Negative Control vs. Positive Control** | -25,29 | -39,22 to -11,35 | Yes | **** | <0,0001 |
| **Negative Control vs. PBM** | 11,62 | -2,311 to 25,56 | No | ns | 0,1434 |
| **Negative Control vs. Clarified Açaí** | 17,80 | 3,872 to 31,74 | Yes | ** | 0,0060 |
| **Negative Control vs. PBM + Clarified Açaí** | 34,62 | 20,69 to 48,56 | Yes | **** | <0,0001 |
| **Positive Control vs. PBM** | 36,91 | 22,98 to 50,84 | Yes | **** | <0,0001 |
| **Positive Control vs. Clarified Açaí** | 43,09 | 29,16 to 57,02 | Yes | **** | <0,0001 |
| **Positive Control vs. PBM + Clarified Açaí** | 59,91 | 45,98 to 73,84 | Yes | **** | <0,0001 |
| **PBM vs. Clarified Açaí** | 6,182 | -7,751 to 20,12 | No | ns | 0,7190 |
| **PBM vs. PBM + Clarified Açaí** | 23,00 | 9,069 to 36,93 | Yes | *** | 0,0002 |
| **Clarified Açaí vs. PBM + Clarified Açaí** | 16,82 | 2,887 to 30,75 | Yes | * | 0,0106 |
| **10 Dias** |  |  |  |  |  |
| **Negative Control vs. Positive Control** | -6,394 | -20,33 to 7,539 | No | ns | 0,6932 |
| **Negative Control vs. PBM** | 8,216 | -5,717 to 22,15 | No | ns | 0,4621 |
| **Negative Control vs. Clarified Açaí** | 28,03 | 14,09 to 41,96 | Yes | **** | <0,0001 |
| **Negative Control vs. PBM + Clarified Açaí** | 48,86 | 34,93 to 62,79 | Yes | **** | <0,0001 |
| **Positive Control vs. PBM** | 14,61 | 0,6772 to 28,54 | Yes | * | 0,0355 |
| **Positive Control vs. Clarified Açaí** | 34,42 | 20,49 to 48,35 | Yes | **** | <0,0001 |
| **Positive Control vs. PBM + Clarified Açaí** | 55,25 | 41,32 to 69,19 | Yes | **** | <0,0001 |
| **PBM vs. Clarified Açaí** | 19,81 | 5,876 to 33,74 | Yes | ** | 0,0017 |
| **PBM vs. PBM + Clarified Açaí** | 40,64 | 26,71 to 54,58 | Yes | **** | <0,0001 |
| **Clarified Açaí vs. PBM + Clarified Açaí** | 20,83 | 6,902 to 34,77 | Yes | *** | 0,0009 |
| **ACAP** |  |  |  |  |  |
| **Tukey's multiple comparisons test** | **Mean Diff.** | **95,00% CI of diff.** | **Significant?** | **Summary** | **Adjusted P Value** |
| **Day 8** |  |  |  |  |  |
| **Negative Control vs. Positive Control** | 46,90 | 32,56 to 61,24 | Yes | **** | <0,0001 |
| **Negative Control vs. PBM** | 26,60 | 12,26 to 40,94 | Yes | **** | <0,0001 |
| **Negative Control vs. Clarified Açaí** | 1,506 | -12,83 to 15,85 | No | ns | 0,9982 |
| **Negative Control vs. PBM + Clarified Açaí** | -33,00 | -47,34 to -18,66 | Yes | **** | <0,0001 |
| **Positive Control vs. PBM** | -20,30 | -34,64 to -5,964 | Yes | ** | 0,0018 |
| **Positive Control vs. Clarified Açaí** | -45,40 | -59,74 to -31,06 | Yes | **** | <0,0001 |
| **Positive Control vs. PBM + Clarified Açaí** | -79,90 | -94,24 to -65,56 | Yes | **** | <0,0001 |
| **PBM vs. Clarified Açaí** | -25,09 | -39,43 to -10,75 | Yes | **** | <0,0001 |
| **PBM vs. PBM + Clarified Açaí** | -59,60 | -73,94 to -45,26 | Yes | **** | <0,0001 |
| **Clarified Açaí vs. PBM + Clarified Açaí** | -34,51 | -48,85 to -20,17 | Yes | **** | <0,0001 |
| **Day 10** |  |  |  |  |  |
| **Negative Control vs. Positive Control** | 19,10 | 4,755 to 33,43 | Yes | ** | 0,0038 |
| **Negative Control vs. PBM** | 15,44 | 1,097 to 29,78 | Yes | * | 0,0290 |
| **Negative Control vs. Clarified Açaí** | -23,67 | -38,01 to -9,328 | Yes | *** | 0,0002 |
| **Negative Control vs. PBM + Clarified Açaí** | -41,90 | -56,24 to -27,56 | Yes | **** | <0,0001 |
| **Positive Control vs. PBM** | -3,658 | -18,00 to 10,68 | No | ns | 0,9505 |
| **Positive Control vs. Clarified Açaí** | -42,76 | -57,10 to -28,42 | Yes | **** | <0,0001 |
| **Positive Control vs. PBM + Clarified Açaí** | -61,00 | -75,34 to -46,66 | Yes | **** | <0,0001 |
| **PBM vs. Clarified Açaí** | -39,10 | -53,44 to -24,76 | Yes | **** | <0,0001 |
| **PBM vs. PBM + Clarified Açaí** | -57,34 | -71,68 to -43,00 | Yes | **** | <0,0001 |
| **Clarified Açaí vs. PBM + Clarified Açaí** | -18,24 | -32,57 to -3,895 | Yes | ** | 0,0063 |
| **Parenchyma area** |  |  |  |  |  |
| **Tukey's multiple comparisons test** | **Mean Diff.** | **95,00% CI of diff.** | **Significant?** | **Summary** | **Adjusted P Value** |
| **Day 8** |  |  |  |  |  |
| **Negative Control vs. Positive Control** | 34472 | 27469 to 41475 | Yes | **** | <0,0001 |
| **Negative Control vs. PBM** | 1846 | -5157 to 8848 | No | ns | 0,9445 |
| **Negative Control vs. Clarified Açaí** | -4227 | -11230 to 2776 | No | ns | 0,4383 |
| **Negative Control vs. PBM + Clarified Açaí** | -9572 | -16575 to -2569 | Yes | ** | 0,0028 |
| **Positive Control vs. PBM** | -32626 | -39629 to -25624 | Yes | **** | <0,0001 |
| **Positive Control vs. Clarified Açaí** | -38699 | -45702 to -31696 | Yes | **** | <0,0001 |
| **Positive Control vs. PBM + Clarified Açaí** | -44044 | -51047 to -37041 | Yes | **** | <0,0001 |
| **PBM vs. Clarified Açaí** | -6073 | -13076 to 929,9 | No | ns | 0,1180 |
| **PBM vs. PBM + Clarified Açaí** | -11417 | -18420 to -4415 | Yes | *** | 0,0003 |
| **Clarified Açaí vs. PBM + Clarified Açaí** | -5345 | -12347 to 1658 | No | ns | 0,2119 |
| **Day 10** |  |  |  |  |  |
| **Negative Control vs. Positive Control** | 33558 | 26555 to 40561 | Yes | **** | <0,0001 |
| **Negative Control vs. PBM** | -7116 | -14119 to -113,2 | Yes | * | 0,0447 |
| **Negative Control vs. Clarified Açaí** | 3554 | -3449 to 10557 | No | ns | 0,6077 |
| **Negative Control vs. PBM + Clarified Açaí** | 2245 | -4758 to 9248 | No | ns | 0,8928 |
| **Positive Control vs. PBM** | -40674 | -47677 to -33671 | Yes | **** | <0,0001 |
| **Positive Control vs. Clarified Açaí** | -30004 | -37007 to -23001 | Yes | **** | <0,0001 |
| **Positive Control vs. PBM + Clarified Açaí** | -31313 | -38316 to -24310 | Yes | **** | <0,0001 |
| **PBM vs. Clarified Açaí** | 10670 | 3667 to 17673 | Yes | *** | 0,0007 |
| **PBM vs. PBM + Clarified Açaí** | 9361 | 2358 to 16364 | Yes | ** | 0,0036 |
| **Clarified Açaí vs. PBM + Clarified Açaí** | -1309 | -8312 to 5694 | No | ns | 0,9839 |
| **Stromal area** |  |  |  |  |  |
| **Tukey's multiple comparisons test** | **Mean Diff.** | **95,00% CI of diff.** | **Significant?** | **Summary** | **Adjusted P Value** |
| **Day 8** |  |  |  |  |  |
| **Negative Control vs. Positive Control** | -116046 | -117393 to -114700 | Yes | **** | <0,0001 |
| **Negative Control vs. PBM** | -50395 | -51741 to -49048 | Yes | **** | <0,0001 |
| **Negative Control vs. Clarified Açaí** | -19769 | -21116 to -18423 | Yes | **** | <0,0001 |
| **Negative Control vs. PBM + Clarified Açaí** | -16394 | -17740 to -15047 | Yes | **** | <0,0001 |
| **Positive Control vs. PBM** | 65651 | 64305 to 66998 | Yes | **** | <0,0001 |
| **Positive Control vs. Clarified Açaí** | 96277 | 94930 to 97623 | Yes | **** | <0,0001 |
| **Positive Control vs. PBM + Clarified Açaí** | 99652 | 98306 to 100999 | Yes | **** | <0,0001 |
| **PBM vs. Clarified Açaí** | 30625 | 29279 to 31972 | Yes | **** | <0,0001 |
| **PBM vs. PBM + Clarified Açaí** | 34001 | 32654 to 35347 | Yes | **** | <0,0001 |
| **Clarified Açaí vs. PBM + Clarified Açaí** | 3376 | 2029 to 4722 | Yes | **** | <0,0001 |
| **Day 10** |  |  |  |  |  |
| **Negative Control vs. Positive Control** | -140611 | -141957 to -139264 | Yes | **** | <0,0001 |
| **Negative Control vs. PBM** | -79042 | -80388 to -77695 | Yes | **** | <0,0001 |
| **Negative Control vs. Clarified Açaí** | -10187 | -11533 to -8840 | Yes | **** | <0,0001 |
| **Negative Control vs. PBM + Clarified Açaí** | 3106 | 1760 to 4453 | Yes | **** | <0,0001 |
| **Positive Control vs. PBM** | 61569 | 60223 to 62915 | Yes | **** | <0,0001 |
| **Positive Control vs. Clarified Açaí** | 130424 | 129078 to 131770 | Yes | **** | <0,0001 |
| **Positive Control vs. PBM + Clarified Açaí** | 143717 | 142371 to 145063 | Yes | **** | <0,0001 |
| **PBM vs. Clarified Açaí** | 68855 | 67509 to 70201 | Yes | **** | <0,0001 |
| **PBM vs. PBM + Clarified Açaí** | 82148 | 80802 to 83494 | Yes | **** | <0,0001 |
| **Clarified Açaí vs. PBM + Clarified Açaí** | 13293 | 11947 to 14639 | Yes | **** | <0,0001 |
| **Acinar area** |  |  |  |  |  |
| **Tukey's multiple comparisons test** | **Mean Diff.** | **95,00% CI of diff.** | **Significant?** | **Summary** | **Adjusted P Value** |
| **Day 8** |  |  |  |  |  |
| **Negative Control vs. Positive Control** | 35610 | 27718 to 43503 | Yes | **** | <0,0001 |
| **Negative Control vs. PBM** | -8281 | -16174 to -388,8 | Yes | * | 0,0354 |
| **Negative Control vs. Clarified Açaí** | -5269 | -13162 to 2624 | No | ns | 0,3364 |
| **Negative Control vs. PBM + Clarified Açaí** | -13053 | -20946 to -5161 | Yes | *** | 0,0002 |
| **Positive Control vs. PBM** | -43892 | -51784 to -35999 | Yes | **** | <0,0001 |
| **Positive Control vs. Clarified Açaí** | -40879 | -48772 to -32987 | Yes | **** | <0,0001 |
| **Positive Control vs. PBM + Clarified Açaí** | -48664 | -56556 to -40771 | Yes | **** | <0,0001 |
| **PBM vs. Clarified Açaí** | 3012 | -4880 to 10905 | No | ns | 0,8158 |
| **PBM vs. PBM + Clarified Açaí** | -4772 | -12665 to 3121 | No | ns | 0,4367 |
| **Clarified Açaí vs. PBM + Clarified Açaí** | -7784 | -15677 to 108,2 | No | ns | 0,0549 |
| **Day 10** |  |  |  |  |  |
| **Negative Control vs. Positive Control** | 29744 | 21851 to 37636 | Yes | **** | <0,0001 |
| **Negative Control vs. PBM** | 4885 | -3007 to 12778 | No | ns | 0,4128 |
| **Negative Control vs. Clarified Açaí** | 6398 | -1495 to 14290 | No | ns | 0,1640 |
| **Negative Control vs. PBM + Clarified Açaí** | -4720 | -12613 to 3173 | No | ns | 0,4479 |
| **Positive Control vs. PBM** | -24858 | -32751 to -16966 | Yes | **** | <0,0001 |
| **Positive Control vs. Clarified Açaí** | -23346 | -31239 to -15453 | Yes | **** | <0,0001 |
| **Positive Control vs. PBM + Clarified Açaí** | -34464 | -42356 to -26571 | Yes | **** | <0,0001 |
| **PBM vs. Clarified Açaí** | 1512 | -6380 to 9405 | No | ns | 0,9824 |
| **PBM vs. PBM + Clarified Açaí** | -9605 | -17498 to -1713 | Yes | ** | 0,0098 |
| **Clarified Açaí vs. PBM + Clarified Açaí** | -11118 | -19010 to -3225 | Yes | ** | 0,0020 |
| **Collagen total area** |  |  |  |  |  |
| **Tukey's multiple comparisons test** | **Mean Diff.** | **95,00% CI of diff.** | **Significant?** | **Summary** | **Adjusted P Value** |
| **Day 8** |  |  |  |  |  |
| **Negative Control vs. Positive Control** | 5176 | 2305 to 8048 | Yes | **** | <0,0001 |
| **Negative Control vs. PBM** | 2863 | -8,783 to 5734 | No | ns | 0,0510 |
| **Negative Control vs. Clarified Açaí** | 1775 | -1097 to 4646 | No | ns | 0,4075 |
| **Negative Control vs. PBM + Clarified Açaí** | -835,6 | -3707 to 2036 | No | ns | 0,9194 |
| **Positive Control vs. PBM** | -2314 | -5185 to 558,0 | No | ns | 0,1657 |
| **Positive Control vs. Clarified Açaí** | -3402 | -6273 to -530,0 | Yes | * | 0,0132 |
| **Positive Control vs. PBM + Clarified Açaí** | -6012 | -8883 to -3140 | Yes | **** | <0,0001 |
| **PBM vs. Clarified Açaí** | -1088 | -3960 to 1784 | No | ns | 0,8146 |
| **PBM vs. PBM + Clarified Açaí** | -3698 | -6570 to -826,8 | Yes | ** | 0,0059 |
| **Clarified Açaí vs. PBM + Clarified Açaí** | -2610 | -5482 to 261,2 | No | ns | 0,0903 |
| **Day 10** |  |  |  |  |  |
| **Negative Control vs. Positive Control** | 5566 | 2694 to 8437 | Yes | **** | <0,0001 |
| **Negative Control vs. PBM** | -614,0 | -3486 to 2258 | No | ns | 0,9726 |
| **Negative Control vs. Clarified Açaí** | -267,4 | -3139 to 2604 | No | ns | 0,9989 |
| **Negative Control vs. PBM + Clarified Açaí** | -2484 | -5356 to 387,2 | No | ns | 0,1179 |
| **Positive Control vs. PBM** | -6180 | -9051 to -3308 | Yes | **** | <0,0001 |
| **Positive Control vs. Clarified Açaí** | -5833 | -8705 to -2961 | Yes | **** | <0,0001 |
| **Positive Control vs. PBM + Clarified Açaí** | -8050 | -10922 to -5178 | Yes | **** | <0,0001 |
| **PBM vs. Clarified Açaí** | 346,6 | -2525 to 3218 | No | ns | 0,9968 |
| **PBM vs. PBM + Clarified Açaí** | -1870 | -4742 to 1001 | No | ns | 0,3548 |
| **Clarified Açaí vs. PBM + Clarified Açaí** | -2217 | -5089 to 654,6 | No | ns | 0,1988 |
